# Structural and functional differences of the thalamus between drug-naïve Parkinson’s disease motor subtypes

**DOI:** 10.3389/fneur.2023.1102927

**Published:** 2023-05-17

**Authors:** Yubing Chen, Zhiying Guo, Yajie Wang, Hangxing Yin, Shugang Zhang, Weiguo Liu

**Affiliations:** Department of Neurology, The Affiliated Brain Hospital of Nanjing Medical University, Nanjing, China

**Keywords:** Parkinson’s disease, functional MRI, thalamus, motor subtypes, drug-naïve

## Abstract

**Objective:**

The thalamus is an integrative hub of motor circuits in Parkinson’s disease (PD). This study aimed to investigate the alterations of structure and functional connectivity (FC) of the thalamic subregions in the tremor-dominant (TD) subtype and the postural instability and gait difficulty (PIGD) subtype in PD.

**Methods:**

A total of 59 drug-naïve patients (24 TD and 35 PIGD) and 37 healthy controls were recruited. The volumes of the thalamus and the thalamic subregions were calculated using FreeSurfer. Functional connectivity (FC) analysis of the resting-state functional MRI (rsfMRI) was conducted on the thalamic subregions. Finally, the altered structure and FC were used for correlation analysis with clinical motor scores and for further motor subtypes differentiation.

**Results:**

The volumes of the left posterior parietal thalamus (PPtha) in TD patients were significantly lower than those of PIGD patients. Compared with PIGD patients, TD patients exhibited higher FC between the thalamic subregions, the left middle temporal gyrus (MTG), the right dorsolateral superior frontal gyrus (SFGdl), the left middle occipital gyrus (MOG), and the right superior temporal gyrus (STG). Compared with HCs, TD patients showed higher FC between the thalamic subregions and the right SFGdl, as well as the left MOG. Compared with HCs, PIGD patients showed lower FC between the thalamic subregions and the left MTG. In addition, the altered FC was closely related to clinical symptoms and performed high-discriminative power in differentiating the motor subtypes.

**Conclusion:**

Increased FC between the thalamic subregions and the sensory cortices in TD patients may indicate a better compensatory capacity for impairment of sensory information integration than that in PIGD patients. The altered FC between the thalamus and the MTG was a potential biomarker for the distinction of the PD motor subtypes.

## Introduction

Parkinson’s disease (PD) is one of the most common neurodegenerative disorders, which is characterized by a broad range of clinical symptoms, including motor and non-motor symptoms (1). Motor symptoms are heterogeneous and individually distinct during the long clinical phase of PD. Thus, motor subtype classifications have been extensively applied in clinical and scientific research. Tremor-dominant (TD)/intermediate/postural instability and gait difficulty (PIGD) subtype classification is the most commonly used because it is convenient, intuitive, and quantifiable (2). This kind of classification identified the two most typical symptoms by using the Unified Parkinson’s Disease Rating Scale (UPDRS). The intermediate type refers to those who could not be accurately differentiated at an early stage of the disease. Compared with the TD subtype, the PIGD subtype is associated with more severe non-motor symptoms, more aggressive disease progression, and a worse prognosis (3–5). However, the neuropathological mechanism behind these differences remains unclear.

The neuropathological mechanism leading to motor symptoms of PD is recognized as a degeneration of dopaminergic neurons in substantia nigra pars compacta (SNc) and dopamine exhaustion at the striatum. Subsequently, these pathological changes cause dysfunction of the striato-thalamo-cortical (STC) circuitry (6). The impairment of the STC circuit was found to be largely responsible for bradykinesia and rigidity symptoms (7, 8). Recent neuroimaging studies on the cerebellar pathways revealed that the cerebello-thalamo-cortical (CTC) circuitry has an important part in tremor etiology (9, 10), which was an addition to the classical motor circuits theory. As an integrative hub of the motor circuits, extensive attention has been focused on the thalamus in the field of PD research. In this regard, an earlier pathological study showed that some of the nuclei of the thalamus lost 30–55% of their neurons in PD patients, which was associated with significant α-synuclein deposition (11). This finding suggests that not only the nigrostriatal system but also thalamic degeneration contribute directly to the development of the disease.

Imaging studies have shown various thalamic abnormalities in different motor subtypes of PD. In a SPECT study, thalamic serotonin transporter density was found to be decreased in early drug-naïve PD patients with tremor (12). Additionally, a resting-state functional MRI (rsfMRI) research indicated that tremor was associated with increased causal connectivity from the ventral intermediate nucleus (Vim) to M1 and the cerebellum, whereas the symptoms of the PIGD subtype were correlated with increased causal connectivity from the Vim to the pre-motor cortex (preM) and the putamen (10). However, no difference was observed in the volumes of the individual thalamic nuclei between the TD and PIGD groups, although several nuclei volumes of patients with each of these two subtypes showed significant differences from the healthy controls (13). It is worth noting that previous studies on the motor subtypes of PD were based on the total thalamus or a single thalamic nucleus. However, it is well known that the thalamus is a functionally heterogeneous brain region, which integrates information from different cortical functional networks. Currently, few studies have been conducted on the functional activity of the thalamic functional subregions in different PD motor subtypes. A recent study on PD patients under medication found increased FC in several thalamic functional subdivisions, suggesting alterations in the basal ganglia-thalamocortical circuitry in PD (14). Nevertheless, the mechanisms of distinct functional activity of the thalamic subregions in different motor subtypes of PD have not been elucidated.

Therefore, in the present study, we applied the Brainnetome Atlas template to divide the thalamus into 16 functional subregions (15). This connectivity-based parcellation structure contained information about both the anatomical and functional connections. We hypothesized that structural and functional differences in certain thalamic subregions would be detected between different PD motor subtypes, and these abnormal changes could contribute to the occurrence of distinct symptoms.

## Materials and methods

### Participants

Participants were consecutively recruited at the Affiliated Brain Hospital of Nanjing Medical University. The diagnosis of PD was made by an experienced neurologist using the United Kingdom Parkinson’s Disease Brain Bank diagnostic criteria (16), and the clinical follow-up period lasted at least 1 year to confirm the diagnosis. The exclusion criteria were as follows: (1) history of taking anti-parkinsonism, anticholinergic, or neuroleptic drugs; (2) history of brain damage, cerebrovascular disorders, seizure, hydrocephalus, intracranial mass, brain operation, severe psychiatric diseases, or other neurologic diseases; (3) dementia; (4) poor MRI image quality or excessive head motion; and (5) an intermediate subtype that could not be defined as TD or PIGD. Additionally, a group of healthy controls (HCs) with normal neurological and psychiatric findings were recruited and matched for age, gender, and education with PD patients. In total, 59 drug-naïve PD patients and 37 HCs were finally included in the study. All participants were right handed. All the participants signed informed consent forms after an explanation of the study details. This study was approved by the Medical Ethics Committee of the Affiliated Brain Hospital of Nanjing Medical University (Nanjing, Jiangsu, China).

### Demographic and clinical data

Demographic data and clinical assessments, including gender, age, years of education, disease duration, Hoehn and Yahr (H&Y) staging scale, and the Unified Parkinson’s Disease Rating Scale (UPDRS), were collected during the baseline visit. All participants were subjected to the cognitive assessment using the Mini-Mental State Examination (MMSE); the MMSE scores of each subject should be ≥24. Patients were classified into a TD and a PIGD group using the UPDRS subscores according to Jankovic’s method (2). The tremor scores were defined as a total number of UPDRS II item 16 and UPDRS III items 20–21. The PIGD scores were defined as a total number of UPDRS II items 13–15 and UPDRS III items 29–30. The ratio of the mean tremor scores to the mean PIGD scores was used to identify the TD (ratio ≥ 1.5) and PIGD (ratio ≤ 1.0) subtypes. Finally, 24 TD patients, 35 PIGD patients, and 37 HCs were included in this study.

### Definition of the thalamus and thalamic subregions

**Figure 1:**
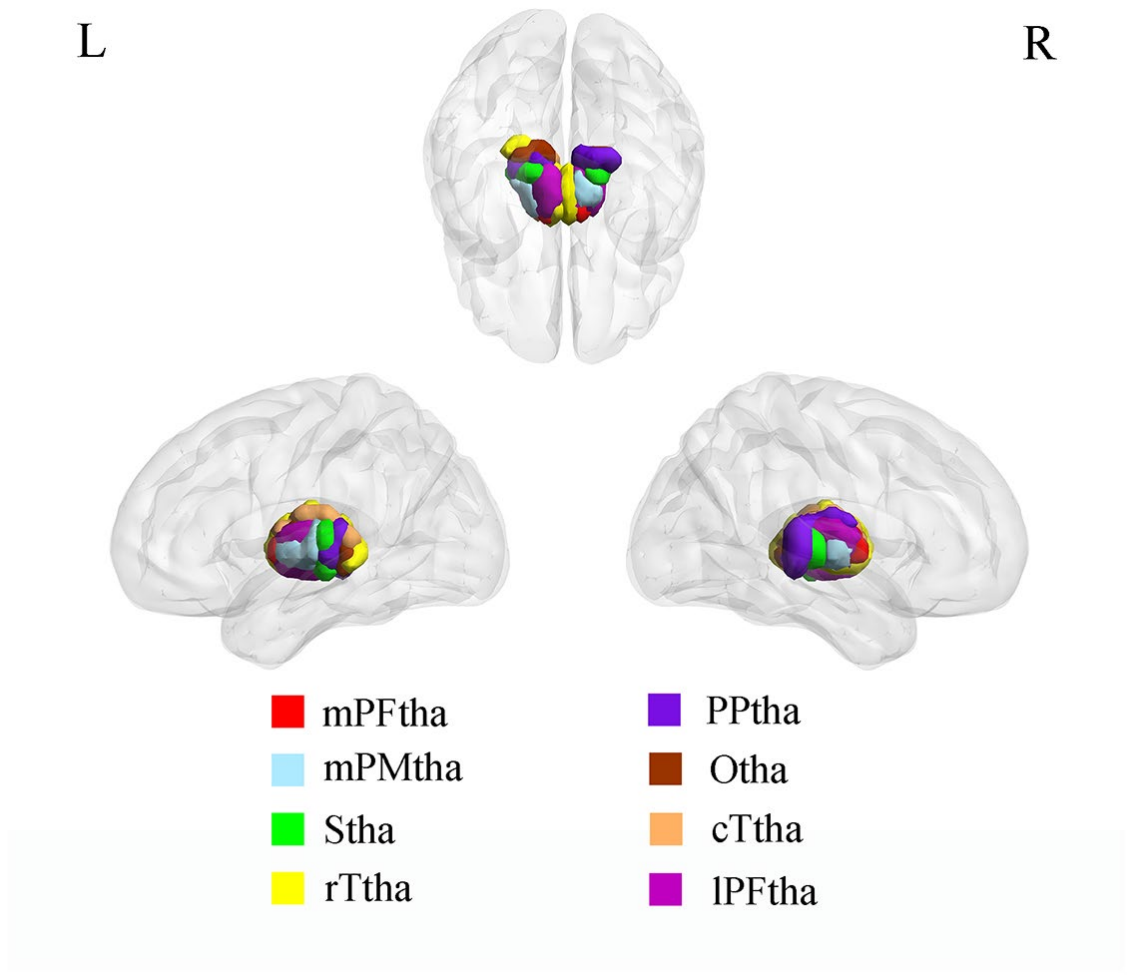
ROI presentation of Thalamic subregions. mPFtha, medial pre-frontal thalamus; mPMtha, pre-motor thalamus; Stha, sensory thalamus; rTtha, rostral temporal thalamus; PPtha, posterior parietal thalamus; Otha, occipital thalamus; cTtha, caudal temporal thalamus; lPFtha, lateral pre-frontal thalamus.

The thalamus and thalamic subregions were defined according to the Brainnetome Atlas, which was a parcellation based on the functional connectional architecture, linking brain connectivity to function (15). The thalamus was divided into 16 subregions, including the medial pre-frontal thalamus (mPFtha), pre-motor thalamus (mPMtha), sensory thalamus (Stha), rostral temporal thalamus (rTtha), posterior parietal thalamus (PPtha), occipital thalamus (Otha), caudal temporal thalamus (cTtha), and the lateral pre-frontal thalamus (lPFtha) (Figure 1). The atlas can be downloaded at http://atlas.brainnetome.org.

### MRI data acquisition

MRI data were acquired using a 3.0 T MRI scanner (Siemens, Verio, Germany) equipped with a standard eight-channel head coil. All participants were required to remain motionless and relaxed with their eyes closed and to stay awake during scanning. T1-weighted images were scanned using a 3D magnetization-prepared rapid gradient-echo (MPRAGE) sequence with the following parameters: repetition time (TR) = 2,530 ms; echo time (TE) = 3.34 ms; matrix = 256 × 192; flip angle (FA) = 7 degrees; field of view (FOV) = 256 mm × 256 mm; number of slices = 128; slice thickness/gap = 1.33/0.5 mm; and bandwidth = 180 HZ/PX. Functional images were obtained with a gradient-recalled echo-planar imaging (GRE-EPI) pulse sequence which contained 240 volumes. The parameters were as follows: TR = 2000 ms; TE = 30 ms; matrix = 64 × 64; FA = 90 degrees; FOV = 220 mm × 220 mm; number of slices = 31; thickness/gap = 3.5/0.63 mm; and bandwidth = 2,232 HZ/PX.

### Image processing

#### Volumetric analysis

The T1-weighted data were preprocessed using FreeSurfer (version 6.0.0)^1^ for registration, segmentation, and measurement of the thalamus and the thalamic subregions’ volumes. The preprocessing procedures were as follows: correction of minor head motion, correction of bias-field inhomogeneity, skull stripping, segmentation of gray and white matter structures and cerebrospinal fluid (CSF), automatic correction of topology defects, smoothing with a 10-mm full-width half-maximum (FWHM) Gaussian kernel, and parcellation of the cortical and subcortical structures into 246 brain regions of the Brainnetome Atlas. All data were visually checked for image integrity and inaccuracy of tissue segmentation. Finally, volumetric measures were calculated using the ratio of the thalamus and the thalamic subregions volumes to the total intracranial volumes (TIV).

#### Functional connectivity analysis

The rs-fMRI data were preprocessed using DPARSF 5.0^2^ (17), which is based on SPM12^3^ in the MATLAB 2013b platform.^4^ The first 10 volumes in the original images were removed for magnetization equilibrium. The remaining 230 volumes were then preprocessed by the following steps: slice timing by aligning the middle slice; head motion correction (participants whose mean framewise displacement (FD) is >0.5 mm or their head motion exceeded a translation of 3 mm or a rotation of 3 degrees were excluded); segmentation of the T1 images into white matter, gray matter, and CSF; normalization into the standard Montreal Neurological Institute (MNI) space using the Diffeomorphic Anatomical Registration Through Exponentiated Lie Algebra (DARTEL) algorithm; resampling to a 3 mm × 3 mm × 3 mm voxel size; temporal band-pass filtering (0.01–0.08 Hz); and spatial smoothing with a full-width half-maximum (FWHM) Gaussian kernel of 6 mm.

The 16 thalamus subregions were defined as regions of interest (ROIs) to perform functional connectivity analysis. The mean time series of these ROIs were extracted. Subsequently, a voxelwise functional connectivity analysis was performed by computing the temporal correlation between these ROIs and the whole brain within the gray matter (GM) mask. The individual correlation coefficients were then converted to z-values using Fisher’s r-to-z transformation. Finally, an entire brain z-value map was obtained for each ROI of each subject.

### Statistical analysis

Statistical analyses were performed using IBM SPSS Statistics (v. 24.0, Armonk, NY, USA). Categorical variables such as gender were compared using the chi-squared test. Kolmogorov–Smirnov test was used to verify whether each variable obeys the normal distribution characteristics. The continuous normally distributed data were analyzed by *t*-test or one-way analysis of variance (ANOVA), otherwise using Mann–Whitney *U*-test or Kruskal–Wallis test. Ages among three groups were compared with a one-way analysis of variance (ANOVA) followed by a *post hoc* two-sample *t*-test. The education level and MMSE scores among groups were analyzed with Kruskal–Wallis test followed by the *post hoc* Mann–Whitney *U*-test with Bonferroni correction. The disease duration, H&Y stage, UPDRS III scores, tremor scores, and PIGD scores of the two PD groups were analyzed with Mann–Whitney *U*-test. ANOVA with the covariates of gender, age, education, and MMSE was implemented to compare the volumes of the total thalamus and 16 thalamic subregions among the three groups. Then *post hoc* two-sample *t*-tests with the covariates of gender, age, education, and MMSE were employed for between-group comparisons. Bonferroni-corrected *p*-value of <0.05 was considered to indicate statistical significance.】 ANOVA was used to detect the significantly different regions in functional connection with each thalamic subregion among the three groups, covarying for gender, age, education, MMSE, and GM volume. A voxelwise threshold of *p* < 0.001 and a clusterwise threshold of *p* < 0.05 with Gaussian random field (GRF)-corrected were considered to indicate statistical significance (18). Then, using these significantly different regions among groups extracted as a mask, *post hoc* two-sample *t*-tests were used to compare thalamic subregions FC values between groups within the mask with covariates of gender, age, education, MMSE, and GM volume. The statistical significance was set to voxelwise threshold of *p* < 0.001 combined with the GRF-corrected clusterwise threshold of *p* < 0.05. Pearson partial correlation analyses were conducted to evaluate the relationships between the altered thalamic subregion volumes or functional connectivity and tremor or PIGD scores with gender, age, education, and MMSE as covariates. Then, based on FC values correlated with both the TD and PIGD scores, simple and multiple binary logistic regression was utilized to investigate the risk factors related to motor subtypes in PD patients. Finally, the predictive value of individual FC indices to differentiate motor subtypes in PD was assessed by the receiver operating characteristic (ROC) curve, and the area under the curve (AUC) values were calculated. A *p*-value of <0.05 was considered to indicate a statistically significant difference.

## Results

### Demographic and clinical data

A total number of 96 participants were enrolled in the present study, including 24 TD patients, 35 PIGD patients, and 37 HCs. The demographic and clinical data of all subjects are summarized in Table 1. There were no significant differences in gender (*p* = 0.914), age (*p* = 0.192), or education (*p* = 0.243) among the three groups. The MMSE scores of the TD and PIGD groups were significantly lower than those of the HCs (*p* < 0.05); however, no significant differences between the two PD subgroups (*p* = 0.772) were identified. Disease duration (*p* = 0.259), H&Y scores (*p* = 0.190), and UPDRS III (*p* = 0.473) were not significantly different between the two PD subgroups.

**Table 1:** Demographic and clinical characteristics of all subjects.

	TD-PD (*N* = 24)	PIGD-PD (*N* = 35)	HC (*N* = 37)	*P* (*P1,P2,P3*) Value
Gender (M/F)	13/11	17/18	19/18	0.914^a^
Age (years)	60.75 ± 8.03	57.49 ± 7.87	59.51 ± 5.05	0.192^b^ (0.095, 0.286, 0.234)
Education (years)	9.17 ± 4.51	10.57 ± 3.40	10.99 ± 3.17	0.243^c^ (0.321, 0.088, 0.483)
MMSE	27.29 ± 1.99	27.17 ± 1.84	28.86 ± 1.29	<0.001^c^,* (0.772, 0.001*,<0.001*)
Duration (years)	2.33 ± 1.61	1.84 ± 1.30	–	0.259^d^
H&Y stage	1.73 ± 0.47	1.57 ± 0.56	–	0.190^d^
UPDRS III	25.38 ± 11.30	23.71 ± 12.14	–	0.473^d^
Tremor scores	7.42 ± 4.39	2.60 ± 1.91	–	<0.001^d^,*
PIGD scores	1.92 ± 1.38	3.17 ± 1.25	–	<0.001^d^,*

Data were presented as mean ± standard deviation; TD-PD, tremor-dominant subtype of PD; PIGD-PD, postural instability and gait difficulty subtype of PD; MMSE, Mini-Mental State Examination; H&Y stage, Hoehn and Yahr stages; UPDRS III, the motor section of the Unified Parkinson’s Disease Rating Scale. *p < 0.05. ^a^Chi-squared test. ^b^ANOVA, followed by post hoc two-sample *t*-test between TD-PD vs PIGD-PD, TD-PD vs HC and PIGD-PD vs HC. ^c^Kruskal–Wallis test, followed by post hoc Mann–Whitney *U*-test with Bonferroni correction. ^d^Mann–Whitney *U*-test.

### Thalamus and thalamic subregions volumes

**Figure 2:**
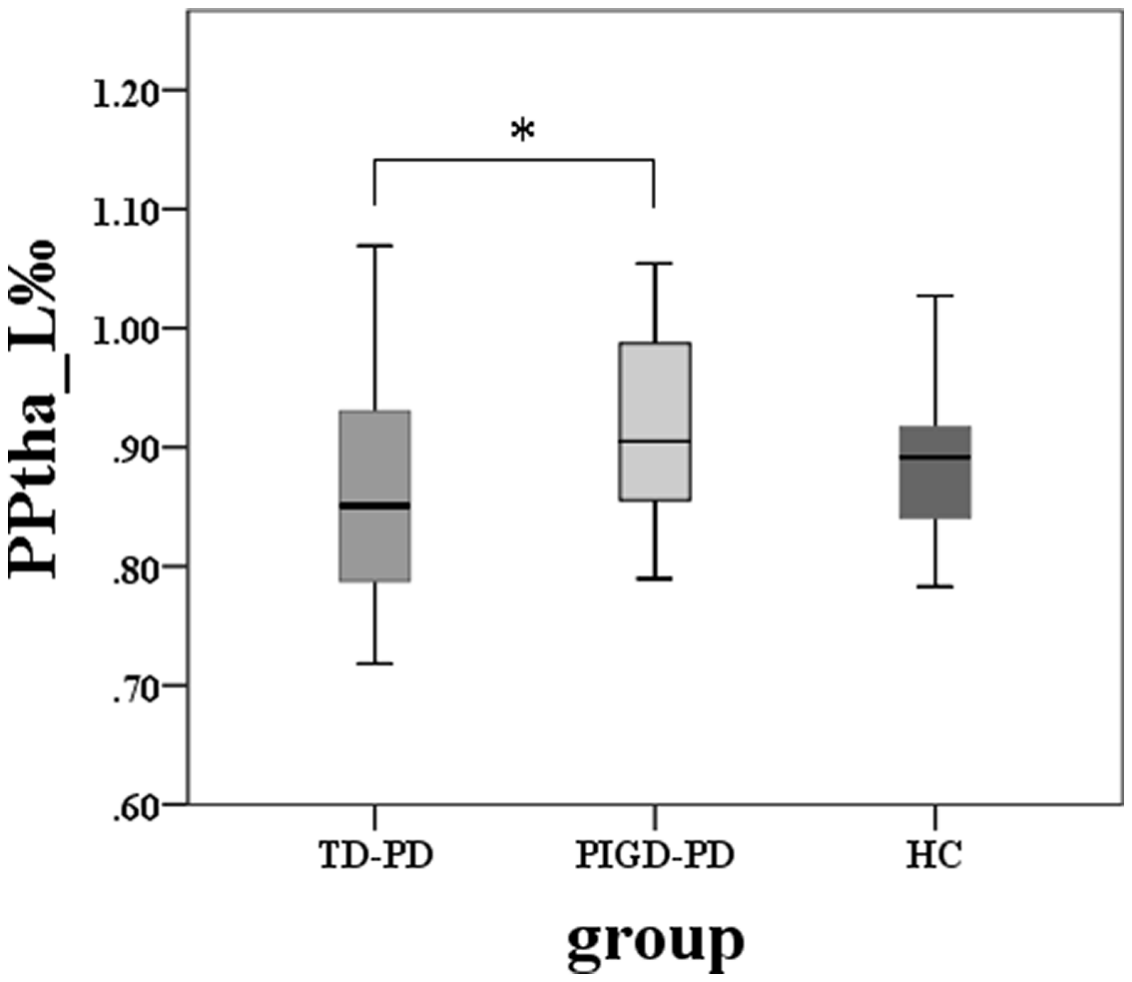
Among-group differences of the total thalamus and thalamic subregions volumes (*two-sample *post hoc t*-tests, *p* < 0.05, Bonferroni-corrected).

The volume of the left PPtha showed significant differences among the three groups (*p* = 0.036), and two groups’ comparisons showed reduced volumes in TD compared with PIGD in the left PPtha (Figure 2). No significant correlations were found between the volumes and tremor or PIGD scores. There were no significant differences in the total thalamus and other thalamic subregion volumes among the three groups.

### Thalamic subregions functional connectivity

#### ANOVA analysis

The ANOVA analysis showed significant FC alterations across the three groups between the right mPFtha, bilateral mPMtha, left Stha, bilateral lPFtha, and the left middle temporal gyrus (MTG); between the left Stha and the right superior frontal gyrus, dorsolateral (SFGdl); and between the right Otha and the left middle occipital gyrus (MOG), as well as the right superior temporal gyrus (STG) (Table 2). The significance threshold of ANOVA was a voxelwise threshold of *p* < 0.001 combined with a GRF-corrected clusterwise threshold of *p* < 0.05.

**Table 2:** Among-group differences of thalamic subregions functional connectivity (voxelwise threshold of *p* < 0.001 combined with a GRF-corrected clusterwise threshold of *p* < 0.05).

Seed area	Cluster size	Connected location (AAL)	BA	MNI coordinate (x, y, z)	Peak T
Right mPFtha	58	Temporal_Mid_L	21	−60 −54 −6	11.936
Left mPMtha	56	Temporal_Mid_L	21	−51 −9 −15	12.008
	56	Temporal_Mid_L	21	−57 −54 −6	16.141
Right mPMtha	60	Temporal_Mid_L	21	−57 −54 −3	17.019
Left Stha	79	Temporal_Mid_L	21	−51 −9 −15	12.953
	181	Frontal_Sup_R	9	15 48 33	12.717
Right Otha	88	Occipital_Mid_L	18	−42 −87 −3	12.565
	48	Temporal_Sup_R	22	60 0 3	12.472
Left lPFtha	46	Temporal_Mid_L	21	−57 −54 −6	15.891
Right lPFtha	79	Temporal_Mid_L	21	−60 −54 −6	17.364

AAL, Anatomical Automatic Labeling; BA, Brodmann area; MNI, Montreal Neurological Institute; mPFtha, medial pre-frontal thalamus; mPMtha, pre-motor thalamus; Stha, sensory thalamus; Otha, occipital thalamus; lPFtha, lateral pre-frontal thalamus; Temporal_Mid_L, left middle temporal gyrus; Frontal_Sup_R, right superior frontal gyrus, dorsolateral; Occipital_Mid_L, left middle occipital gyrus; Temporal_Sup_R, right superior temporal gyrus.

##### *Post-hoc* test

**Figure 3:**
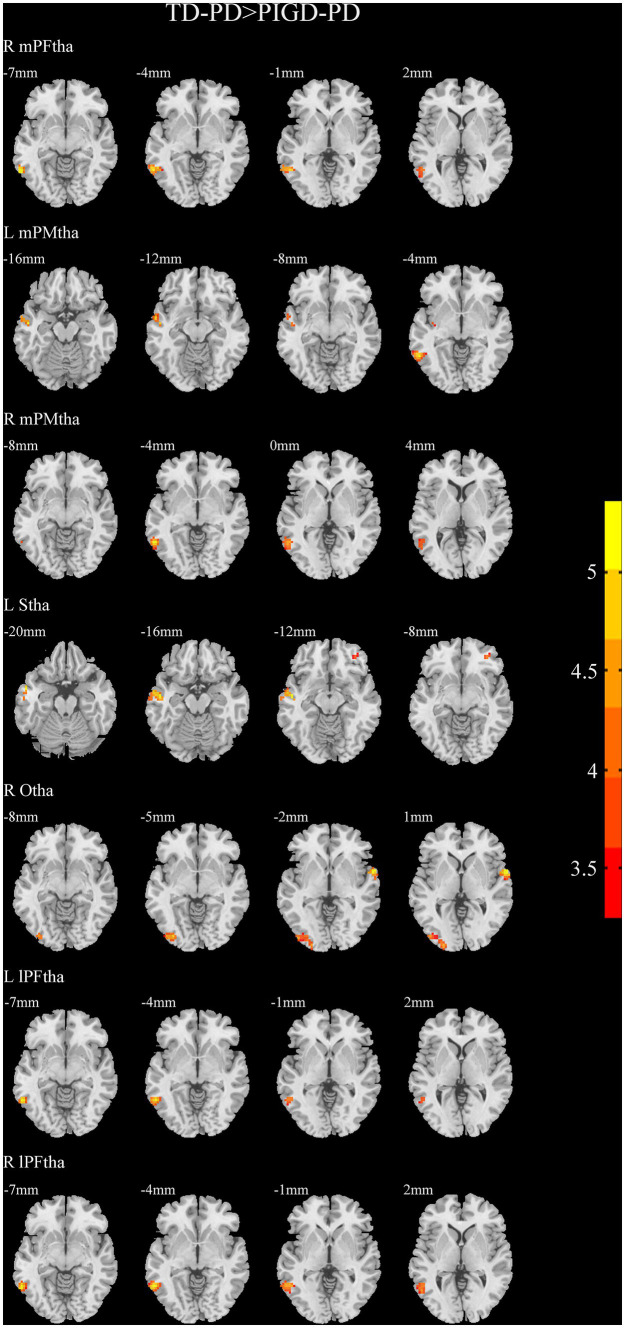
Results of two-sample *t*-test between TD patients and PIGD patients. All results are in MNI space, the red color represents the increased FC, while the blue color represents the decreased FC (voxelwise threshold of *p* < 0.001 combined with a GRF-corrected clusterwise threshold of *p* < 0.05).

**Figure 4:**
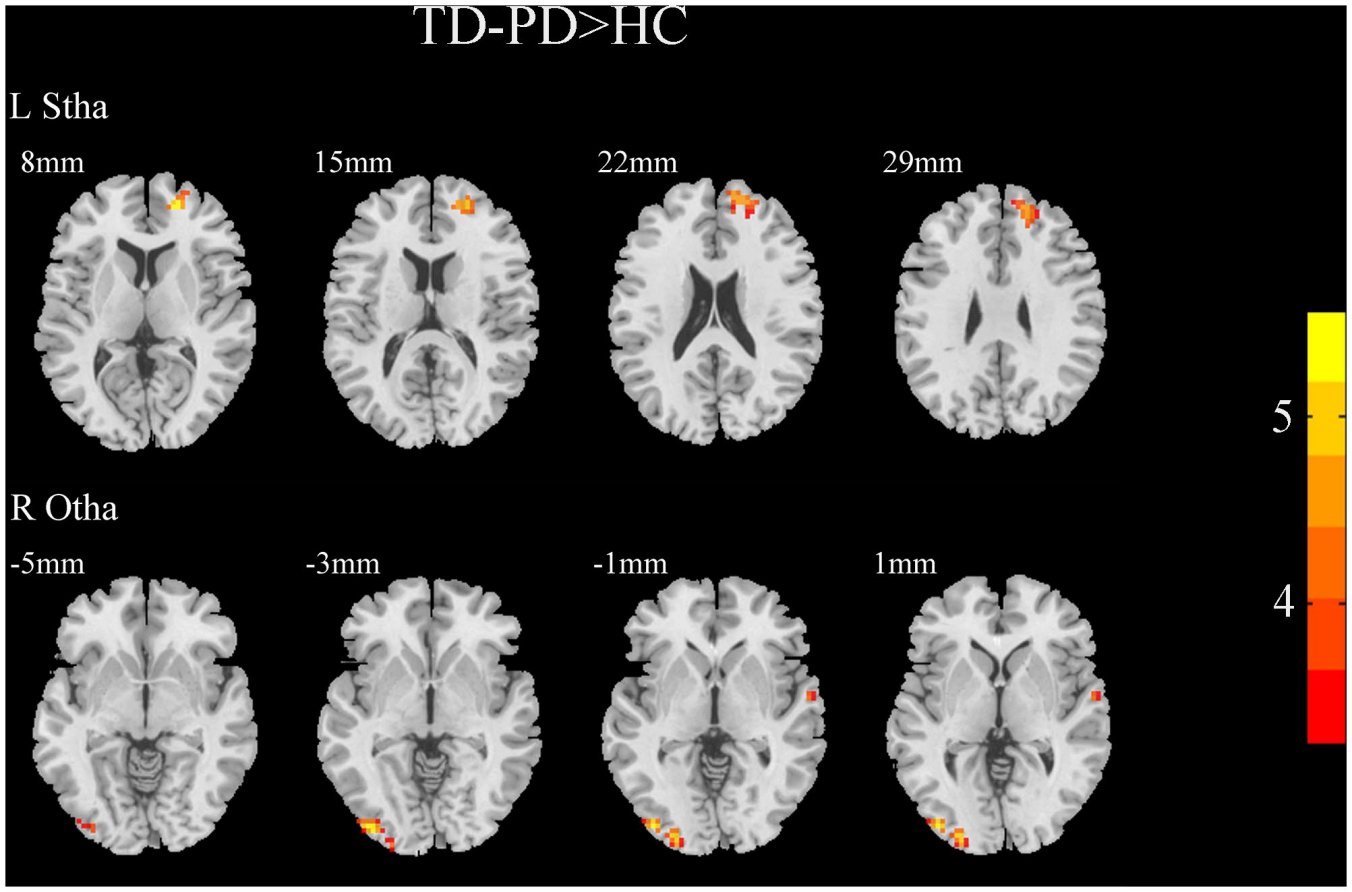
Results of two-sample *t*-test between TD patients and HCs. All results are in MNI space, the red color represents the increased FC, while the blue color represents the decreased FC (voxelwise threshold of *p* < 0.001 combined with a GRF-corrected clusterwise threshold of *p* < 0.05).

**Figure 5:**
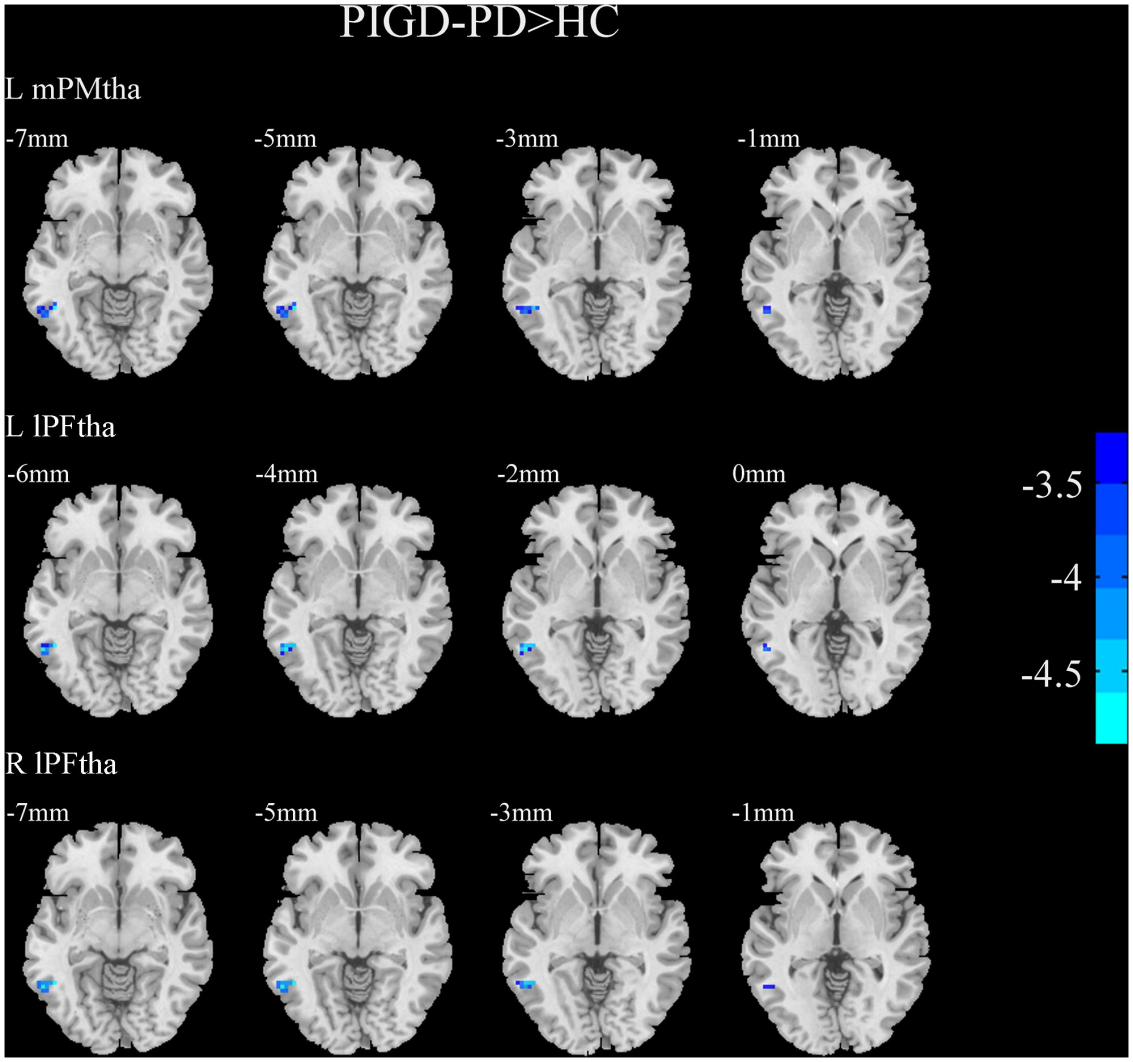
Results of two-sample *t*-test between PIGD patients and HCs. All results are in MNI space, the red color represents the increased FC, while the blue color represents the decreased FC (voxelwise threshold of *p* < 0.001 combined with a GRF-corrected clusterwise threshold of *p* < 0.05).

A two-sample *t*-test of the differences between the TD and PIGD patients showed that the TD group had higher FC between the right mPFtha, bilateral mPMtha, left Stha, bilateral lPFtha, and the left MTG; between the left Stha and the right SFGdl; and between the right Otha and the left MOG, as well as the right STG (Figure 3). The FC between left Stha and the right SFGdl and between the right Otha and the left MOG in the TD group was higher than that in the HC group (Figure 4). The PIGD group had lower FC between the left MTG and the left mPMtha, as well as bilateral lPFtha than the HC group (Figure 5).

### Correlation analysis

**Figure 6:**
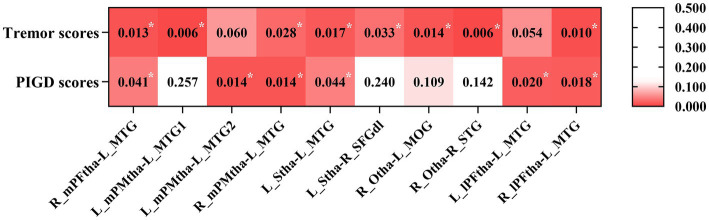
The correlation between the altered FC and clinical data in PD patients with gender, age, education, and MMSE as covariates. Asterisks indicate a significant correlation (*p* < 0.05).

To explore the behavioral significance of the altered FC in thalamic subregions, we conducted partial correlation analyses between the significantly altered FC with tremor and PIGD scores. The results showed that tremor scores were positively correlated with the FC between the right mPFtha, bilateral mPMtha, the left Stha, the right lPFtha, and the MTG; between the left Stha and the right SFGdl; and between the right Otha and the left MOG, as well as the right STG. The PIGD scores were negatively correlated with FC between the right mPFtha, bilateral mPMtha, left Stha, bilateral lPFtha, and the left MTG (Figure 6).

### Binary logistic regression analysis

Based on the results of correlation analysis, we included FC values between the left MTG and the right mPFtha, the right mPMtha, the left Stha, and the right lPFtha in the simple binary logistic regression analysis. The results showed independent associations of the PD motor subtypes with these FC values. Multiple binary logistic regression with stepwise selection showed that FC values between the left Stha and the left MTG were independent indicators of PD motor subtypes (Table 3).

**Table 3:** Univariate and multivariate logistic regression analysis for the factors influencing motor subtype differentiation of PD.

Variables	Univariate			Multivariate		
	OR	95%CI	*P-*value	OR	95%CI	*P-*value
FC of R_mPFtha-L_MTG	0.003	0.000–0.085	0.001			
FC of R_mPMtha-L_MTG	<0.001	0.000–0.028	0.001			
FC of L_Stha-L_MTG	<0.001	0.000–0.008	<0.001	<0.001	0.000–0.145	0.012
FC of R_lPFtha-L_MTG	0.000	0.000–0.038	0.001			

OR, odds ratio; CI, confidence interval.

### Receiver operating characteristic curve analysis for the diagnosis of Parkinson’s disease patients with different motor subtypes

**Figure 7:**
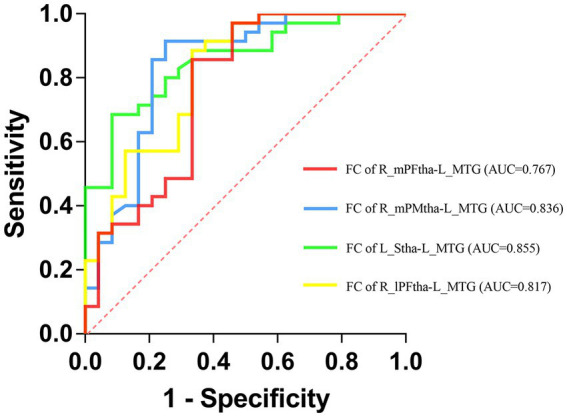
Receiver operating characteristic (ROC) curves for differentiating TD patients from PIGD patients by neuroimaging biomarkers.

We selected the FC values between the left MTG and the right mPFtha, the right mPMtha, the left Stha, and the right lPFtha as biomarkers to differentiate TD patients from PIGD patients based on the results of the regression analysis. We found that the area under the curve (AUC) values were 0.767 (95%CI = 0.635–0.899, *p* = 0.001) for FC between the right mPFtha and the left MTG, 0.836 (95%CI = 0.723–0.950, *p* < 0.001) for FC between the right mPMtha and the left MTG, 0.855 (95%CI = 0.761–0.950, *p* < 0.001) for FC between the left Stha and the left MTG, and 0.817 (95%CI = 0.704–0.930, *p* < 0.001) for FC between the right lPFtha and the left MTG (Figure 7).

## Discussion

In this study, we investigated structural and functional differences in the thalamic subregions between the motor subtypes of drug-naïve PD patients. The analysis of morphology revealed reduced volumes in the left PPtha in TD patients compared with PIGD patients. Our fsMRI findings showed significantly higher FC between the left MTG, the right SFGdl, the left MOG, the right STG, and corresponding thalamic subregions in TD patients than in PIGD patients. These functional alterations were closely related to the manifestations of clinical symptoms. In addition, we established that FC is a potential biomarker that can distinguish PD motor subtypes by the application of the ROC curve analysis.

In this study, we found that the volumes of the left PPtha in TD patients were lower than those in PIGD patients. However, no correlation between the volumes of this region and clinical symptoms was observed. In our subsequent research, we found no FC difference in the left PPtha between the two motor subtypes. Therefore, it seems that the functional changes or clinical symptoms in this type of thalamic subregion atrophy cannot be explained. Studies on the thalamic volumes of PD subtypes have yielded inconsistent results. Earlier investigations showed no difference in the total thalamic volumes between PD motor subtypes (19, 20). However, a recent study revealed differences in the volumes of several thalamic nuclei between the two PD subtypes and the presence of a distinct intrinsic thalamic structural local network between them (13). We suggest that anatomically based subcortical nuclei segmentation would be more suitable for thalamic volumetric analysis than functionally based subregions. In addition, with the development of imaging techniques, ultra-high field MR technology would be able to distinguish more subtle differences in their subcortical structure that could not be currently examined.

One of our most important findings is the significantly higher FC between the left MTG and thalamic seeds in TD patients than in PIGD patients, including the right mPFtha, bilateral mPMtha, left Stha, and bilateral lPFtha. In addition, compared to HCs, TD patients showed increased FC, while PIGD patients showed decreased FC in these areas, although not statistically significant. The MTG participates in various functional activities, such as movement observation, language processing, and logical reasoning (21). Previous neuroimaging studies have established that MTG is involved in cognitive impairment in PD (22, 23), but few of them have mentioned how MTG is involved in the motor circuits and causes motor symptoms. MTG is the main region of the human middle temporal complex (MT+), which has been evidenced to play a vital role in visual motion perception in humans. Visual motion information was found to be conveyed from the retina via the lateral geniculate nucleus (LGN) of the thalamus through the primary visual cortex (V1) to the MT+, the core area for visual motion processing (24). PD patients were observed to have reduced activation in MT+ during optic flow processing, indicating that dysfunction in visuoperceptual cognition of PD may be caused by impaired MT+ function (25). PD is known to be related to the impairment of visual perception ability and vision-based activities in daily life. In this regard, non-tremor PD patients had significantly worse visual activities of daily living, including light/dark adaptation and depth perception, than tremor patients (26). Gait disturbance in PD was linked to worse vision functions and spatial cognition (27, 28). Thus, in agreement with the findings of the abovementioned studies, our results of decreased thalamus–MTG connection in PIGD patients provided neuroimaging evidence for worse visual motion perception and vision-based behavior in this subtype than those in TD patients. In addition, although there was no significant difference between the two PD groups and HCs, we suppose there was still a trend of compensation in TD patients and decompensation in PIGD patients. The limited difference may result from the fact that the patient groups were in the early stage of the disease and therefore could not be distinguished from HCs. Then, we found that the changed FC of the MTG and most thalamic subregions were positively correlated with tremor scores and negatively correlated with PIGD scores. According to the behavioral results of the Brainnetome Atlas, the right mPFtha, the right mPMtha, and the bilateral lPFtha are associated mostly with perception, whereas the left mPMtha and the left Stha are associated with action and execution (15). Hence, in this study, the increased FC of the MTG in TD patients was suggested to be compensated for impairment of visual motion perception at an early stage of the disease, whereas the decreased FC in PIGD patients represented damaged visual motion processing function and might contribute to postural and gait impairment in this motor subtype. Our findings support better treatment and physiotherapy exercise aiming at visual processing defects in early-stage PD patients, especially in the PIGD subtype.

The SFGdl highly corresponds to the dorsolateral part of the dorsolateral pre-frontal cortex (dlPFC, Brodmann area 9) (29), a region for generating sensory information and planning future motor events (30). Previous neuroimaging research has shown that dlPFC of the akinetic/rigid motor subtype patients had reduced activation and greater gray matter loss than that of TD patients (31, 32). Similarly, we found that FC between the left Stha and the right SFGdl in TD patients was significantly higher than that in PIGD patients and HCs. In addition, according to the behavioral results of the Brainnetome Atlas, the left Stha is associated with action and execution (15). Therefore, we speculate that our results represented compensatory enhancement or an adaptive response of motion information integration and action control impairment in the TD subtype. In contrast, the loss of compensatory capacity for these dysfunctions in PIGD patients might give rise to worse performance in gait disorders and balance impairment.

The STG processes auditory signals from the primary auditory cortex and then via the thalamus sends them to higher-order areas and other language areas (33). The thalamo-temporal junction is closely related to the processing and integration of auditory information input. PD patients on dopaminergic medication were evidenced to have higher activity in the right STG during auditory beat-omission processing than HCs, indicating that there were compensatory mechanisms for temporal auditory information processing in PD (34). In this study, TD patients exhibited higher FC between the right STG and the right Otha than PIGD patients, whereas no significant difference was observed between the two groups and HCs in this regard. We consider that this result might be due to the fact that the included patients were at the early stage of the disease and had not received dopaminergic treatment. Thus, no difference existed in the brain activity between these patients and HCs with these confounding factors. However, it is worth noting that our results suggested that PIGD patients had worse functional activities in terms of auditory signal input and information integration than TD patients during the early disease stage.

We also found that TD patients had higher FC between the left MOG and the right Otha than PIGD patients and HCs. The occipital lobe is known as a center of visual processing have been extensively reported to be associated with visual hallucinations in PD (35). Inconsistent results were obtained in investigations of PD subtypes using functional neuroimaging methods. One study showed that the regional homogeneity (ReHo) of MOG in PIGD patients was lower than that in HCs (36), whereas another reported the opposite result (37). FC between the subthalamic nucleus and MOG in PIGD patients was significantly higher than that in TD patients and HCs (38). The results of the aforementioned studies and the findings of this research confirm in agreement the abnormal functional activity of MOG in PD, indicating visual processing impairment. As for the difference in the results, we consider that visual information processes are affected by visual perception, visuospatial function, and cognition level. Therefore, in our subsequent studies on visual brain regions, we will probably include specific cognitive domain scores, such as those for visuospatial processing, action, and executive function, for further comparative analysis.

The ROC analysis showed that the altered FC between the left MTG and the right mPFtha, the right mPMtha, the left Stha, and the right lPFtha generated high accuracy of estimation, suggesting that the FC between the MTG and the thalamus could serve as an effective biomarker to differentiate TD patients from PIGD patients. Therefore, we assume that PD motor subtypes should be further investigated on visual signal processing in future studies to develop early diagnostics in this respect. Considering that in recent years, increasingly more differences have been found among PD subtypes, which have been attributed to the heterogeneity of various motor subtypes, such as LRRK2 and GBA mutations, CSF concentrations of beta-amyloid 1–42, tau, and alpha-synuclein (39–41), we will incorporate these biomarkers into our cohort study for better evaluation and discrimination between motor subtypes in our further studies.

Finally, we have to acknowledge that the present study had several limitations. First, the study sample size was relatively small and the 1-year follow-up time was still short for PD diagnosis, which has inevitably contributed to some inaccuracy in our results. Second, although the subjects we recruited were all cognitively unimpaired, there were statistical differences in the MMSE scores between patients and HCs. This led to the effect of cognition as a confounding factor on brain functional activity differences between HCs and the other two groups. Therefore, we included MMSE scores as a covariate to lessen its effect. Finally, a deficiency of the image template might have occurred due to the functional parcellation and the absence of matching of the functional subregions with specific thalamic nuclei. Hence, our results lack comparability with the ones of several previous investigations on the functional connectivity of the thalamic nuclei. However, the main advantage of the present research was that the template provided the behavioral domains and paradigm classes of each subregion, which were then subjected to analyses of functional significance.

## Conclusion

In summary, the altered FC between the sensory cortices (the MTG, SFGdl, STG, and MOG) and the thalamic subregions may reflect impaired visual motion perception, auditory signal processing, and sensory information integration in drug-naïve PD patients. In addition, FC can be an effective biomarker for the differentiation of TD patients from PIGD patients. Our findings provide further evidence for the presence of functional activity differences in the thalamic subregions between drug-naïve PD patients of TD and PIGD subtypes. Furthermore, the present results contribute to explaining the origin of the heterogeneity in different clinical phenotypes, thus providing novel insights into new therapeutic targets and differential treatments for patients with different motor subtypes.

## Data availability statement

The raw data supporting the conclusions of this article will be made available by the authors, without undue reservation.

## Ethics statement

The studies involving human participants were reviewed and approved by the Medical Research Ethics Committee of the Affiliated Brain Hospital of Nanjing Medical University. The patients/participants provided their written informed consent to participate in this study.

## Author contributions

SZ and WL designed and organized the research and made important revisions to the manuscript. YC, ZG, YW, and HY collected the imaging and assessment scale data. YC and ZG were responsible for formal analysis. YC drafted the manuscript. All authors contributed to the manuscript and approved the submitted version.

## Funding

This work was supported by the National Key Research and Development Program of China (2017YFC1310302 and 2016YFC1306600), the National Natural Science Foundation of China (81571348, 81903589, and 81701671), and the Science and Technology Program of Jiangsu Province (BE2019611).

## Conflict of interest

The authors declare that the research was conducted in the absence of any commercial or financial relationships that could be construed as a potential conflict of interest.

## Publisher’s note

All claims expressed in this article are solely those of the authors and do not necessarily represent those of their affiliated organizations, or those of the publisher, the editors and the reviewers. Any product that may be evaluated in this article, or claim that may be made by its manufacturer, is not guaranteed or endorsed by the publisher.

## Footnotes

^1^ http://surfer.nmr.mgh.harvard.edu

^2^ http://rfmri.org/DPARSF

^3^ http://www.fil.ion.ucl.ac.uk/spm

^4^ http://www.mathworks.com/products/matlab
